# Understanding eating-related health outcomes: connections between anxiety and eating behavior

**DOI:** 10.1093/eurpub/ckac129.698

**Published:** 2022-10-25

**Authors:** FS Hussenoeder, I Conrad, M Löbner, M Löffler, A Tönjes, M Sturmvoll, A Villringer, S Riedel-Heller

**Affiliations:** 1 ISAP, Leipzig University, Leipzig, Germany; 2 IMISE, Leipzig University, Leipzig, Germany; 3 Medical Department III, Leipzig University, Leipzig, Germany; 4 Human Cognitive and Brain Sciences, Max-Planck-Institute, Leipzig, Germany

## Abstract

**Background:**

Research shows that anxiety is connected to a variety of mental health outcomes, and that it is widespread among the population. In the light of the great personal and societal costs of obesity and eating disorders, we want to understand the connection between anxiety and different dimensions of eating behaviors that have a strong empirical link with negative eating-related health outcomes.

**Methods:**

We used data from the population- based LIFE-Adult-Study (n = 5019) to analyze the connection between anxiety (GAD-7) and the three dimensions of eating behaviors: Cognitive Restraint, Disinhibition, and Hunger (FEV, German version of the Three-Factor-Eating-Questionnaire). We controlled for sociodemographic variables, smoking, physical activity, personality, and social support.

**Results:**

Multivariate regression analyses showed significant positive associations between anxiety and Disinhibition (β = 0.23, p ≤ 0.001), Hunger (β = 0.21, p ≤ 0.001) and Cognitive Restraint (β = 0.04, p ≤ 0.01). After adding control variables, analyses revealed significant positive associations between anxiety and Disinhibition (β = 0.15, p ≤ 0.001) as well as Hunger (β = 0.14, p ≤ 0.001), but not between anxiety and Cognitive Restraint (β = 0.03, p = 0.076).

**Conclusions:**

There is an empirical connection between anxiety and two factors of eating behavior, i.e., Disinhibition and Hunger. If future research strengthens the assumption of a causal direction from anxiety to those factors, interventions that help individuals to better regulate and cope with anxiety, could be one potential pathway to reducing eating disorders and obesity in the population.

**Key messages:**

• There is a significant connection between anxiety and eating behavior.

• Interventions that address anxiety could reduce problematic eating-related health outcomes like eating disorders and obesity.

